# Deceptive measures of progress in the NHS long-term plan for cancer: case-based vs. population-based measures

**DOI:** 10.1038/s41416-023-02308-9

**Published:** 2023-06-17

**Authors:** Jason L. Oke, Sarah Jo Brown, Chris Senger, H. Gilbert Welch

**Affiliations:** 1 Nuffield Department of Primary Care Health Sciences, Oxford University, Oxford, England; 2 Manchester, NH USA; 3 The Center for Surgery and Public Health, Department of Surgery, Brigham and Women’s Hospital, Boston, MA USA

**Keywords:** Cancer, Population screening

## Abstract

The NHS Long Term Plan for cancer aims to increase early-stage diagnoses from 50% to 75% and to have 55,000 more people each year survive their cancer for at least 5 years following diagnosis. The targets measures are flawed and could be met without improving outcomes that really matter to patients. The proportion of early-stage diagnoses could increase, while the number of patients presenting at a late-stage remains the same. More patients could survive their cancer for longer, but lead time and overdiagnosis bias make it impossible to know whether anyone had their life prolonged. The target measures should switch from biased case-based measures to unbiased population-based measures that reflect the key objectives in cancer care: reducing late-stage incidence and mortality.

## Introduction

In June 2018, the UK Prime Minister announced a new 5-year funding settlement for The National Health Service (NHS) in return for developing a long-term plan for the service. One of the goals of the NHS Long Term Plan is “to save thousands more lives each year by dramatically improving how we diagnose and treat cancer”. [1] In January 2019, Health secretary Matt Hancock set out two key 2028 targets as a means to achieve this goal:

1. *The proportion of all cancers diagnosed at an early stage would rise from approximately 50% currently to 75%*.
2. *55,000 more people each year would survive their cancer for at least 5 years following diagnosis*.

These targets would be achieved by implementing a series of initiatives, including an overhaul and expansion of existing cancer screening programmes, the introduction of new tests, mobile lung cancer screening units and significant investment in artificial intelligence (AI) to better target at-risk populations.

While we applaud the goal, the target measures are flawed. While these targets could be achieved through meaningful improvements for patients with cancer, they could also be met without making a single improvement in the outcomes that really matter to patients: a reduced risk of suffering symptoms from cancer or a reduced risk of dying from cancer. Furthermore, the pursuit of these targets could even harm patients directly, by diagnosing and treating cancers that were otherwise not destined to cause problems, and indirectly, by siphoning resources away from more effective health initiatives.

The problem is in the target measures themselves. Both stage distribution and 5-year survival are case-based measures—that is, both use the number of diagnosed cancer cases in the denominator (Table 1). Here we show how both can be deceptive in signalling apparent benefit when none exists. We argue that progress against cancer must be measured using a population-based denominator—specifically, late-stage incidence and mortality.

**Table 1:** NHS target measures, definitions, problems and alternatives.

	Stage distribution	Survival
NHS 2028 target	*The proportion of all cancers diagnosed at an early stage would rise from approximately 50% currently to 75%*	*55,000 more people each year would survive their cancer for at least 5 years following diagnosis*
Target measure [case-based denominator]	Rising % early stage: $\frac{{{{{{{\rm{No.}}}}}}\;{{{{{\rm{of}}}}}}\;{{{{{\rm{early}}}}}} \mbox{-} {{{{{\rm{stage}}}}}}\;{{{{{\rm{cancers}}}}}}}}{{{{{{{\rm{all}}}}}}\;{{{{{\rm{cancers}}}}}}\;{{{{{\rm{diagnosed}}}}}}}}$	Rising 5-year survival: $\frac{{{{{{{\rm{No.}}}}}}\;{{{{{\rm{alive}}}}}}\;5\;{{{{{\rm{years}}}}}}\;{{{{{\rm{after}}}}}}\;{{{{{\rm{diagnosis}}}}}}}}{{{{{{{\rm{all}}}}}}\;{{{{{\rm{cancers}}}}}}\;{{{{{\rm{diagnosed}}}}}}}}$
Problem	Will rise with additional detection of early-stage cancers, even if the number of late-stage cancers remains unchanged	Will always rise with early detection because of lead time bias. If substantial overdiagnosis occurs, survival will markedly rise while mortality remains unchanged
Unbiased alternative [population-based denominator]	Declining Late-stage incidence: $\frac{{{{{{{\rm{No.}}}}}}\;{{{{{\rm{of}}}}}}\;{{{{{\rm{late}}}}}} \mbox{-} {{{{{\rm{stage}}}}}}\;{{{{{\rm{cancers}}}}}}}}{{{{{{{\rm{Population}}}}}}}}$	Declining Mortality: $\frac{{{{{{{\rm{No.}}}}}}\;{{{{{\rm{of}}}}}}\;{{{{{\rm{cancer}}}}}}\;{{{{{\rm{deaths}}}}}}}}{{{{{{{\rm{Population}}}}}}}}$

Note: Population denominators are typically defined as the number of people living in a geographic area (e.g. US, England, etc.) at mid-year. They may be further restricted to the population at risk (e.g. males for prostate cancer, females for cervical cancer). The resulting rates should either be restricted to specific age groups or adjusted for the age structure of the population. (In a randomised trial of screening, the population refers to the study populations: those randomised to screening and those randomised to usual care.)

## Cancer paradigms: the traditional view

Diagnosing cancer earlier is a goal sought by individuals, health systems and governments across the world. The rationale is familiar: cancers found at an early stage are apparently more “curable” and require less aggressive treatment—with fewer attendant side effects.

**Fig. 1:**
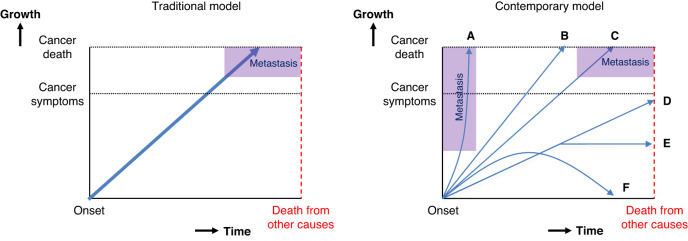
Two models of cancer progression. The traditional model (left) posits that all cancers are destined to follow an orderly progression from the primary site, to the lymph nodes, on to distant metastatic sites, ultimately causing cancer death. The contemporary model (right) is more complex and heterogeneous. Some cancers are metastatic at their onset (A), some never metastasise, yet cause death from local invasion (B), while others follow the traditional model (C). Still other cancers are not destined to ever cause symptoms because they either: grow so slowly that patients die from other causes before symptoms appear (D), stop growing (E) or regress (F).

This strategy makes sense under a widely-held model of cancer progression typically attributed to William Stewart Halsted [2]. Halsted argued that cancer progresses in an orderly fashion: it arises at a single location, grows there, and then eventually spreads to other parts of the body (Fig. 1, left-hand panel). Crucially, in terms of early detection, this model posits that cancer metastasis only happens *late* in the disease, many years after the onset of cancer. Furthermore, this homogeneous model of progression suggests that all cancers, if left untreated, will relentlessly progress to ultimately metastasise and cause death. Under the traditional model, it follows that finding more early-stage cancers is always beneficial.

## Cancer paradigms: the contemporary view

The traditional model is outdated. It is far too simple to adequately represent the constellation of diseases currently labelled as “cancer” [3]. The contemporary model of cancer progression is necessarily more complex and heterogeneous (Fig. 1, right-hand panel).

In the 1960s and 70s, Bernard Fisher questioned Halsted’s view of orderly cancer progression. He hypothesised that breast cancer could be a systemic disease from the outset: that tumour cells could be disseminated throughout the body by the time of detection [4]. Recent cancer genomic research suggests Fisher’s hypothesis extends beyond breast cancer. In an analysis of 118 biopsies from 23 colorectal cancer patients with distant metastases, dissemination was estimated to occur well before the primary tumour was large enough to be clinically detectable [5]. These aggressive, “born to be bad” cancers would elude any feasible early detection efforts, yet they are the ones most likely to cause death.

Cancers at the opposite extreme of the growth spectrum became apparent with the advent of widespread prostate cancer screening in the United States during 1990s. Some localised prostate cancers grew so slowly that they were not destined to causes symptoms before the patient died from competing risks of death—particularly in older men [6, 7]. Alternatively, some lesions meeting the pathological criteria for cancer may not grow at all. The same phenomena soon became evident in randomised trials of chest X-ray screening for lung cancer [8]. Adding to the complexity were subsequent observations suggesting that some breast [9], thyroid [10] and kidney [11] cancers, in fact, regress. Collectively, the detection of these very slow growing, non-progressive, and regressing cancers became known as overdiagnosis—the diagnosis of a “disease” not otherwise destined to be experienced by the patient.

We are only beginning to learn about the heterogeneity of cancer growth. But it seems likely that this heterogeneity exists within cancer primary sites. In other words, there are some breast, colorectal and lung cancers that are already systemic by the time they are detectable and there are others that are not destined to ever metastasise. Under the contemporary model, it follows that finding more early-stage cancer is not always beneficial—and, in fact, can be harmful.

## How stage distribution can be deceptive

*“The proportion of all cancers diagnosed at an early stage would rise from approximately 50% currently to 75%. “*

The contemporary model acknowledges that some early-stage cancers are not destined to become late-stage cancer. Thus, it is possible to find more early-stage cancers yet have no effect on the number of individuals who first present with late-stage cancer. Nonetheless, the case-based measure of stage-distribution will become apparently more favourable simply by finding more early-stage disease.

**Fig. 2:**
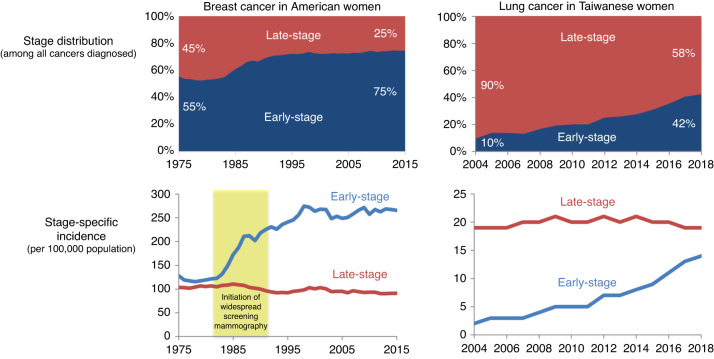
Deceptive stage distributions. Two examples in which a more favorable stage-distribution was largely the result of more early-stage detection, not less late-stage cancer presentation [12, 13].

Two prominent examples of this phenomenon appear in Fig. 2. The introduction of widespread screening with mammography in the United States during the 1980s led to many more breast cancers being detected at an early stage, while the incidence of late-stage breast cancer remained about the same [12]. Nevertheless, the stage-distribution became apparently more favourable: before screening 55% of breast cancers were diagnosed at an early stage, after screening 75% were diagnosed at an early stage. The reframed statement is arguably more powerful: before screening 45% of breast cancers were diagnosed at a late stage, while after screening 25% were diagnosed at a late stage. Yet both statements are deceptive as there was little change in incidence of late-stage disease.

A similar pattern was recently observed with the promotion of low-dose computed tomography lung cancer screening in Taiwanese women—the majority of whom have never smoked [13]. Many more lung cancers were detected at an early stage, while the incidence of late-stage lung cancers remained stable. Again, the stage distribution became apparently more favourable: before screening 90% of lung cancers were diagnosed at a late stage, while after screening 58% were diagnosed at a late stage. These two examples highlight how a favourable change stage distribution can be deceptive and why a shift in stage distribution does not by itself provide evidence that patients have benefited.

## How survival can be deceptive

*“55,000 more people each year would survive their cancer for at least 5 years following diagnosis”*

Even under the traditional model of cancer progression, it is possible to find cancers earlier yet have no effect on when patients die from their cancer—simply because treatment initiated earlier conferred no advantage over treatment initiated later. Nevertheless, earlier detection biases the case-based measure of survival time. Because survival time is measured from the time of diagnosis, cancer screening will always “start the clock earlier”—thus always lengthen survival times. Whether life is prolonged (that is, death is delayed) is a separate question. In the simplest case—no change in the time of death—survival time will lengthen and signal a benefit when none exists. Yet even if death has been delayed, survival time will exaggerate the apparent effectiveness of screening. Because of this so-called lead time bias [14], higher survival does not necessarily mean that earlier detection has prolonged patient’s lives.

**Fig. 3:**
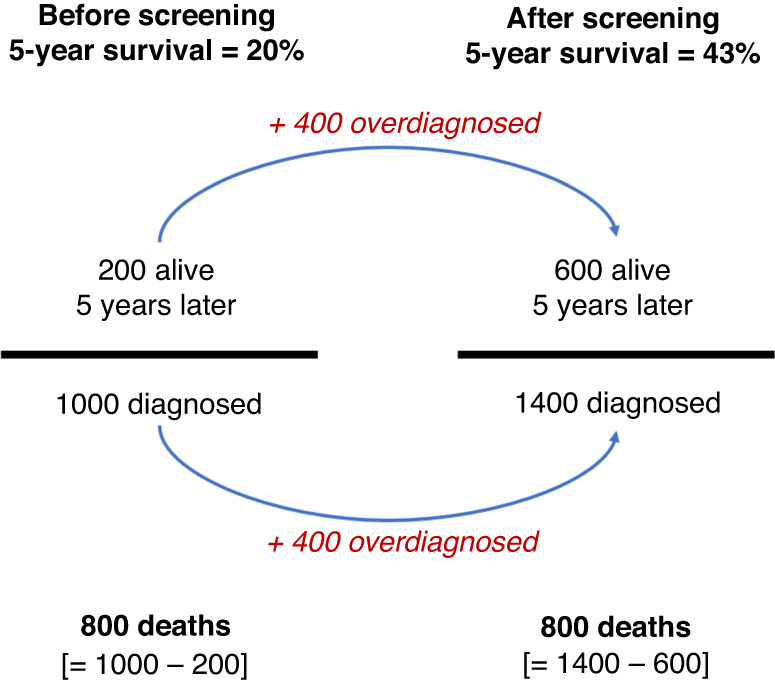
Illustration of how overdiagnosis inflates 5-year survival, while the number of deaths remains unchanged. Overdiagnosed cancers (those cancers not destined to cause death) increase both the numerator and denominator of the 5-year survival statistic—thus causing the proportion to rise, despite no change in the number of deaths. The data shown approximate the change in lung cancer 5-year survival observed in Taiwanese women from 2004 to 2013 [13].

But there is another, potentially larger bias associated with contemporary model of cancer progression: the detection of cancers not destined to cause symptoms or death. The introduction of screening tends to uncover these sub-clinical cancers that have previously gone unnoticed. Overdiagnosis wreaks havoc on survival statistics (Fig. 3).

The scale of this problem should not be underestimated. For example, when fee-for-service providers introduced thyroid screening with ultrasonography in South Korea, the incidence of thyroid cancer increased 15 times over a decade. All of the increase consisted of small papillary thyroid cancers—long known to be a common finding at autopsy, but an extremely rare cause of death [15]. More than 40,000 people were diagnosed with the disease in 2011 alone—virtually all of whom survive 5 years or more. In fact, a website promoting Korean medical tourism advertised Korea as the place be treated for thyroid cancer—touting “the highest thyroid cancer survival rate in the world” [16].

There is no evidence anyone benefited from screening, but many were certainly harmed by unneeded surgery and loss of thyroid function. Yet by these actions, South Korea, a country with a smaller population than the UK, very nearly managed to get 55,000 more people each year surviving their cancer for at least 5 years following diagnosis simply by screening for thyroid cancer.

While survival is a perfectly valid measure in a randomised trial of treatment, survival comparisons across time (e.g. 1980 vs today) or place (e.g. UK vs. US) may say more about diagnostic practice than the quality of treatment or the risk of death [17]. In thyroid cancer, for example, 5-year survival is 87% in the UK and 98% in the US [18, 19]. While it is tempting to imagine thyroid cancer treatment must be better in the US, thyroid cancer mortality is actually lower in the UK (2.4 vs 3.0 per million age-standardised to the world population) [20].

## Moving forward—population-based measures

The NHS target measures, stage distribution and survival, regularly overstate the value of early cancer detection. The problem with these *case*-based measures is that early detection efforts influence both the numerator and the denominator, making it impossible to discern whether genuine progress has been made. What is needed is a stable denominator—one unaffected by early detection—the population (Table 1).

### Late-stage incidence

Declining late-stage cancer incidence suggests that screening is doing what it is intended to do: advance the time of diagnosis for cancers otherwise destined to present clinically at a late-stage. It is important to emphasise that late-stage incidence only includes patients in whom the cancer is *first* diagnosed at a late stage; it does not include those in whom cancer is diagnosed at an early stage, but nonetheless progress to a late stage [21]. Cancers destined to clinically present at a late stage represent the most aggressive and deadly cancers. They are the ones we most want to find early, in the hope that treatment initiated earlier will confer some benefit over treatment initiated later.

Declining late-stage incidence may not lead to fewer deaths, however, because treatment initiated earlier is not reliably more effective than treatment initiated later. The UKCTOCS ovarian cancer screening trial, for example, was able to reduce late-stage (Stage IV) incidence by 25%, yet this earlier detection and treatment did not translate into fewer ovarian cancers deaths [22]. The authors explanation for this was that “the cancers shifted to an earlier stage had an intrinsic poor prognosis”—in other words, they were born to be bad. Randomised trials of breast [23] and colon cancer [24] surveillance showed similar results: aggressive surveillance did detect cancer recurrence earlier, yet earlier detection and treatment did not change the risk of death. Thus, while a reduction late-stage incidence is evidence that screening works in terms of advancing the time of diagnosis for the worst cancers, it does not necessarily mean that patients are being helped.

### Mortality: all causes vs target cancer

“The risk of death is the risk with which the individual is most concerned”, said Sir Richard Doll 30 years ago, when examining whether progress was being made on cancer [25]. It is still true today: reduced mortality remains the most important measure of progress against cancer.

The language is subtle but unambiguous: it is the risk of death from all causes that concerns patients, not simply the risk of dying from cancer. Averting death from cancer only to succumb to some other cause is not really progress—some have even argued that dying from other causes may be worse [26].

Randomised trials of screening for lung [27], colon [28], and prostate cancer [29] have demonstrated that screening significantly reduced the risk of dying from the target cancer but had no impact on all-cause mortality. The apparent paradox may be the result of both (1) off-target deaths (i.e. deaths that are a consequence of screening and subsequent intervention, yet are not ascribed to the target cancer) and (2) the competing risks of death associated with the ageing soma (i.e. those at a high risk of dying from cancer are also a high risk of dying from other causes) [30]. Patients and NHS policymakers learning that screening “saves lives” might reasonably expect that screening would enhance their longevity (i.e. reduce all-cause mortality). But that may not be the case.

Alternatively, the apparent paradox may be explained more simply: as being the result of the play of chance. All-cause mortality is an insensitive measure for population wide interventions targeting a single cancer (e.g. colon or lung cancer) as deaths from the target cancer are a small component of all deaths. A trial screening for one cancer powered to detect the effect on all deaths would require a Herculean effort—hundreds of thousands of people followed for a decade or more. Thus as the NHS looks to lower the starting age for colon cancer screening (from age 60 years to age 50 years) or expand lung cancer screening by adding mobile units it is reasonable to measure progress in terms of colon or lung cancer mortality. But as the NHS considers interventions intended to address all cancers combined—such as AI to better target at-risk populations and multi-cancer early detection tests (liquid biopsies)—we would argue not only is reduced all-cause mortality the best measure of progress, but also that it is an achievable one, as all cancers combined are a substantial component of all deaths [31].

## Conclusion

Death is not the only outcome relevant to early cancer detection, other outcomes matter as well. It is conceivable, for example, that earlier detection might reduce the symptom burden of some cancer patients without extending their life. But it is far more likely that screening produces additional burden for others. First, many healthy people have to be persuaded that they “need” to be tested—too often with scary messages suggesting that people who die from cancer could have avoided the outcome with earlier detection. Then there are the problems caused by abnormal results: the emotional and psychological stress in those falsely alarmed, the routine subsequent testing of those deemed to be at “high risk” because of a detected abnormality, and the toxicity and complications of unneeded treatment in those overdiagnosed.

The conundrum of cancer screening is that while only a few participants can potentially benefit, all can be potentially harmed. Thus, arguments for more screening require that its benefit be sufficiently large to warrant the associated harms and opportunity costs. As we have shown here, surrogate measures of benefit can be deceptive—what is required is evidence that screening, in fact, saves lives. This will be hard to do because the effect being sought is necessarily small. Given the evolving understanding that tumour biology and host response are more relevant to prognosis than the time of diagnosis, we believe it’s time to challenge the assertion that more screening is the best strategy to make progress against cancer.

## Data Availability

Data sharing not applicable to this article as no data sets were generated or analysed during the current study.
